# Evolution of *pfhrp2* and *pfhrp3* deletions in Equatorial Guinea between the pre– and post–RDT introduction

**DOI:** 10.1186/s12936-024-05036-4

**Published:** 2024-07-18

**Authors:** Irene Molina-de la Fuente, M. Andreína Pacheco, Luz García, Vicenta González, Matilde Riloha, Consuelo Oki, Agustín Benito, Ananias A. Escalante, Pedro Berzosa

**Affiliations:** 1 Biomedicine and biotechnology Department, University of Alcalá, Ctra.Madrid-Barcelona Km.33,600, 28871 Alcalá de Henares, Spain; 2 National Centre of Tropical Medicine, Carlos III Institute of Health, C/ Sinesio Delgado 10, 28029 Madrid, Spain; 3 Consorcio Centro de Investigación Biomédica en Red – CIBERINFEC ISCIII, C/ Sinesio Delgado 10, 28029 Madrid, Spain; 4 Biology Department/Institute of Genomics and Evolutionary Medicine (iGEM), Temple University, (SERC - 645), 1925 N. 12 St, Philadelphia, PA 19122-1801 USA; 5 Ministry of Health and Social Welfare (MINSABS), National Programne for Malaria Control, Malabo, Equatorial Guinea

**Keywords:** Malaria, RDT, False negatives, Africa, Subgenus* Laverania*

## Abstract

**Background:**

*Pfhrp2* and *pfhrp3* deletions are threatening *Plasmodium falciparum* malaria diagnosis by rapid diagnostic tests (RDT) due to false negatives. This study assesses the changes in the frequencies of *pfhrp2* and *pfhrp3* deletions (*pfhrp2*^*Del*^ and *pfhrp3*^*Del*^, respectively) and the genes in their flaking regions, before and after RDT introduction in Equatorial Guinea.

**Methods:**

A total of 566 *P. falciparum* samples were genotyped to assess the presence of *pfhrp2* and *pfhrp3* deletions and their flanking genes. The specimens were collected 18 years apart from two provinces of Equatorial Guinea, North Bioko (Insular Region) and Litoral Province (Continental Region). Orthologs of *pfhrp2* and *pfhrp3* genes from other closely related species were used to compare sequencing data to assess *pfhrp2* and *pfhrp3* evolution. Additionally, population structure was studied using seven neutral microsatellites.

**Results:**

This study found that *pfhrp2*Del and *pfhrp3*Del were present before the introduction of RDT; however, they increased in frequency after their use, reaching more than 15%. Haplotype networks suggested that *pfhrp2*Del and *pfhrp3*Del emerged multiple times. Exon 2 of *pfhrp2* and *pfhrp3* genes had high variability, but there were no significant changes in amino acid sequences.

**Conclusions:**

Baseline sampling before deploying interventions provides a valuable context to interpret changes in genetic markers linked to their efficacy, such as the dynamic of deletions affecting RDT efficacy.

**Supplementary Information:**

The online version contains supplementary material available at 10.1186/s12936-024-05036-4.

## Background

Global malaria control efforts are stalled. There were 249 million cases in 2022, more than 90% in Africa, where *Plasmodium falciparum* is the most prevalent species [1]. Progress toward malaria elimination is contingent on having to prompt, affordable, and sensitive diagnosis.

The World Health Organization (WHO) recommends parasitological confirmation to receive anti-malarial treatment [2]. Although light microscopy remains the gold standard, rapid diagnostic tests (RDT) targeting *P. falciparum* histidine-rich protein 2 (*Pf*HRP2) protein and its cross-reactive histidine-rich protein 3 (PfHRP3) protein are widely used in sub-Saharan African countries due to their affordability, easy performance, and interpretation [2]. These proteins are abundant in parasite blood stages, but their function is unclear [3]. RDTs were introduced in Equatorial Guinea (West-Central Africa) in 2012 but have been more widely used in Bioko Island, where malaria control efforts have been scaled up, leading to lower prevalence. In contrast, there are less intense control measurements in the Continental region, leading to higher malaria prevalence [4].

*Pf*HRP2 and *Pf*HRP3 are encoded by subtelomeric genes located in chromosomes 8 and 13, respectively. Such areas are prone to deletions. Indeed, reports of RDTs false negatives associated with *pfhrp2* and *pfhrp3* gene deletions (*pfhrp2*^*Del*^ and *pfhrp3*^*Del*^*,* respectively) are increasing worldwide, threatening the use of RDTs [3]. *pfhrp2*^*Del*^ and *pfhrp3*^*Del*^ have been reported in Central African countries, including Equatorial Guinea [5–8]. Single deletion (*pfhrp2*^*Del*^ or *pfhrp3*^*Del*^) could lead to RDT positive diagnosis, as the remaining protein could be still detected [3]. Deletions of linked genes could be present upstream and downstream [9], reaching 40% of flanking deletions in Africa [10, 11]. However, patterns and extension of block deletions are still unclear [9, 12].

Clonal expansions of *pfhrp2*^*Del*^ lineages have been reported in Peru [13], Ethiopia [14], Eritrea [15], and the China—Myanmar border [16]. This is interpreted as driven by selection due to RDT use and “test—treat” strategies. Contrastingly, there is no evidence of selection in *pfhrp3*^*Del*^ [14]. Independent multiple origins have been reported for *pfhrp2*^*Del*^ and *pfhrp3*^*Del*12^ [14, 15]. Although its impact is unclear [17], amino acidic sequence variability in the *Pf*HRP2 and *Pf*HRP3 proteins may also affect RDT sensibility [18, 19]. Thus, understanding the dynamic of *pfhrp2*^*Del*^ and *pfhrp3*^*Del*^ locally is critical to assessing RDT’s long-term efficacy.

This study describes and compares the changes in *pfhrp2*^*Del*^ and *pfhrp3*^*Del*^ prevalence before and after RDT introduction in Equatorial Guinea, considering two provinces with differences in their malaria control interventions. The origins and patterns of deletions, including flanking genes of *pfhrp2* and *pfhrp3,* were explored using a comparative approach with other non-human malaria species. The evolution of exon 2 of *pfhrp2* and *pfhrp3* genes was also characterized using phylogenetic analysis.

## Methods

### Study site and sample collection

This study included samples separated by, at least, 18 years from two provinces of Equatorial Guinea: North Bioko, (Insular Region) and Litoral Province (Continental region) (Table 1; Fig. S1). Samples from North Bioko were collected from symptomatic children under 5 years old for 2001 and 2018 [20, 21], and samples from Litoral Province were from symptomatic children under 5 years old for 2001 samples [22], and from the asymptomatic general population for 2019 [22]. Whole blood was collected from finger pricks and preserved as dried blood spots (DBS) using Whatman 903TM paper. The DBS were stored in double zip-lock plastic bags with silica gel at − 20 °C, and while they were being used, they were stored at 4 °C.

**Table 1:** Sample information

Region	Province	Year	Nº samples	Age range
Island	North Bioko	1999–2001	127	0–5
Island	North Bioko	2018	131	0–5
Continental	Litoral	2001	113	0–5
Continental	Litoral	2019	195	5–66

### Ethical statement

Samples were taken from patients, or their parents or caregivers after they signed informed consent, guarded in the Ministry of Health and Social Welfare of Equatorial Guinea (MINSABS), and transferred to the National Centre for Tropical Medicine (CNMT-Spain) (C.0005278/C.0005279/C.0005485).

### DNA extraction and detection of ***pfhrp2***^***Del***^ and ***pfhrp3***^***Del***^

DNA was extracted from DBS using the Saponin/Chelex method [23]. Confirmation of *P. falciparum* infections where performed using the malaria PCR assay [23]. Independent amplification of two fragments, one including exon 1 and the beginning of exon 2 and the other including exon 2, of both *pfhrp2* and *pfhrp3* genes were performed by semi-nested or conventional PCR as previously described [23]. In the absence of amplification, the PCR was repeated two more times to confirm deletion. *Plasmodium falciparum* 3D7 clone (intact *pfhrp2* and *pfhrp3* genes) was used as a positive control, clone Dd2 (*pfhrp2*^*Del*^) as a negative control for the *pfhrp2* gene amplification, and clone HB3 (*pfhrp3*^*Del*^) as a negative control for the *pfhrp3* gene amplification. Finally, the amplification of single-copy genes *pfdhps* and *pfdhfr* was used as a control to ensure a sufficient amount/quality of DNA.

### Flanking regions of *pfhrp2* and *pfhrp3* genes and data analysis

Genes flanking *pfhrp2* (PF3D7_0831900 and PF3D7_0831700) and *pfhrp3* (PF3D7_1372100 and PF3D7_1372400) genes were amplified according to Akinyi et al. [12]. Haplotypes of each sample were reconstructed by combining information on the *pfhrp2* or *pfhrp3* genes and their respective flanking genes. Comparisons between frequencies were calculated using an X^2^ or Fisher’s exact test depending on the number of samples included in each group. P-values of < 0.05 were considered statistically significant. All data were analysed using R v4.0.

### Comparative study of *pfhrp2* and *pfhrp3 *genes in subgenus *Laverania*

*Pfhrp2* and *pfhrp3* orthologous genes from *Plasmodium* species from subgenus *Laverania* were identified and downloaded using BLAST tool [24] in PlasmoDB [25]. Synteny of flanking *pfhrp2* and *pfhrp3* regions of the different species were analysed using JBrowse in PlasmoDB [25]. Available sequences of *pfhrp2* and *pfhrp3* genes were aligned using the ClustalX (v2.0.12), implemented in SeaView (v4.3.5) software with manual editing [26]. Phylogenetic relationships were inferred using both Bayesian method implemented in MrBayes [27] and a Maximum Likelihood method in IQ-TREE [28]. Then, phylogenetic trees were visualized and edited using the FigTree (v1.4.3) software [29].

### Population genetic analyses

Genotyping was performed using fluorescently labelled PCR primers for amplification of seven *P. falciparum* standardized neutral microsatellite loci following previously published conditions [12, 30] (Table S1). Amplified fragments were multiplexed and separated on AB3730 XL DNA Analyzer (Applied Biosystems) by capillary electrophoresis. All the alleles for each locus with more than 200 relative fluorescence units (rfu) were scored using Geneious software. Peaks lower than one-third the height of the predominant peak were discarded [31]. Infections with more than one allele at any locus were considered multiple infections, and infections with only one allele per locus at all loci were considered single infections. No amplification was reported as missing data and not considered for defining the multiplicity of infection.

Nei’s index of genetic diversity (*He*), defined as *He* = [*n*/(*n* − 1)][1 − ∑] [32], was estimated for each locus and considering all for each group of samples using Haplotype Analysis software v1.04 [33]. *He* gives the average probability that a pair of alleles randomly selected from the population is different.

Population structure was analysed using a Bayesian model-based clustering approach- to infer the number of genetically related cluster or populations (*K*) based on the seven neutral microsatellite profiles obtained. Structure v.2.3.4 software was used with 430 samples (with only one or zero missing values) that have single infection or multiple infections with only one locus with more than one allele, so their haplotype can be faced, there were 56 and 147 from 2001 to 2018 respectively, from North Bioko, and 91 and 136 from 2001 to 2019 respectively from Litoral province. The admixture model was used in all analyses, allowing the presence of samples with ancestry in more than one of the *K* populations [34]. To assess the most likely K—number for the data, Delta K values were calculated using Structure Harvester v0.6.94, K value with higher delta K were then considered as the best clustering for the data [35]. Results for the most likely K—populations were interpreted using CLUMPP (Cluster Matching and Permutation Program) [36] and graphically displayed using *distruct v1.1* [37].

Haplotype genealogies were estimated using an Minimum Spanning Tree—like structure inferred for the seven neutral microsatellites using the Global Optimal eBURST algorithm [38] implemented in PHYLOViZ software [39]. Haplotypes were compared according to province, year of collection, and *pfhrp2*^*Del*^ or *pfhrp3*^*Del*^ status.

### Amino acid variation in *pfhrp2* and *pfhrp3* genes

Amplified products of exon 2 of *pfhrp2* and *pfhrp3* genes were purified using Ilustra Exoprostar 1-step (GE Healthcare Life Sciences) and sequenced using a standard dye terminator in an ABI PRISM 3730 XL Analyser (Big Dye Terminator v3.1 Cycle Sequencing kit) as was mentioned before. All samples were sequenced in both directions, using forward and reverse primers (6 pmol/μL) and sequences from samples with single infections were overlapped using Bio Edit Sequence Alignment Editor Software v7.1.3.0. Final sequences were then translated into amino acids. Analysis of the frequency and number of occurrences of each amino acid repeat [18, 40] or epitope [19] was carried out with a custom code using Python 3.9 Software [23]. That analysis was repeated for available sequences of subgenus *Laverania*.

## Results

### Frequency of*** pfhrp2***^***Del***^ and ***pfhrp3***^***Del***^ exon 2 per locality

First, *pfhrp2*^*Del*^ and *pfhrp3*^*Del*^ frequencies of the total 566 samples were analysed independently for each province, focusing on temporal changes, and then both provinces were compared to understand geographical differences. *pfdhps* and *pfdhfr* genes for all samples were successfully amplified.

**Fig. 1:**
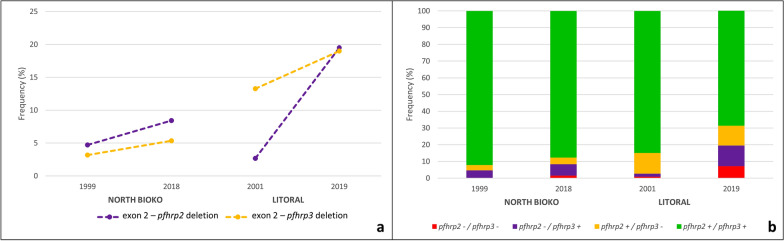
Frequencies of *pfhrp2*^*Del*^ and *pfhrp3*^*Del*^ per sampled year and location. **a** The dotted lines are used to show the increase of the *pfhrp2*^*Del*^ and *pfhrp3*^*Del*^ per locality. **b** Frequency of combination of the presence and/or *pfhrp2*^*Del*^ + *pfhrp3*^*Del*^

In North Bioko, *pfhrp2*^*Del*^ and *pfhrp3*^*Del*^ frequencies increased from 1999 to 2018 (Table S2). Deletion of exon 2 of both genes reached more than 5% in 2018 (Fig. 1a). However, according to *X*^*2*^–test, the increase was only significant for *pfhrp3*^*Del*^ (p–value < 0.05). Parasites conserving both genes were the most common over time, and the frequency of double–deletions or *pfhrp2*^*Del*^ + *pfhrp3*^*Del*^ was maintained low (Fig. 1b; Table S3).

Significant increases of *pfhrp2*^*Del*^ and *pfhrp3*^*Del*^ were observed in Litoral region, using *X*^*2*^–test (Fig. 1a). Deletion frequency of exon 1–2 and 2 of both genes reached 15% in 2019 (Table S2). Although, conservation of both genes was the most common genotype over the years, frequencies of single *pfhrp2*^*Del*^ and double—(*pfhrp2*^*Del*^ + *pfhrp3*^*Del*^) significantly increased in 2019 (Fig. 1b; Table S3).

Notably, before RDT introduction, the *pfhrp2*^*Del*^ frequency was similar in North Bioko and Litoral provinces whereas *pfhrp3*^*Del*^ was higher in the Litoral Province (Table S2). Although in both provinces *pfhrp2*^*Del*^ and *pfhrp3*^*Del*^ increased after introducing RDTs, their increase in Litoral province was higher in *pfhrp2*^*Del*^ and *pfhrp3*^*Del*^ genes (Fig. 1). Regarding exon 2—deletion combinations, the frequencies were also higher in Litoral than in Bioko province, where single *pfhrp2*^*Del*^ and double *pfhrp2*^*Del*^ + *pfhrp3*^*Del*^ markedly increased, but only the increase in the single *pfhrp2*^*Del*^ was significant (Table S3). Single *pfhrp3*^*Del*^ was maintained at a relatively high frequency before and after RDT introduction (Fig. 1).

### Haplotypes flanking ***pfhrp2***^***Del***^ and ***pfhrp3***^***Del***^***.***

**Fig. 2:**
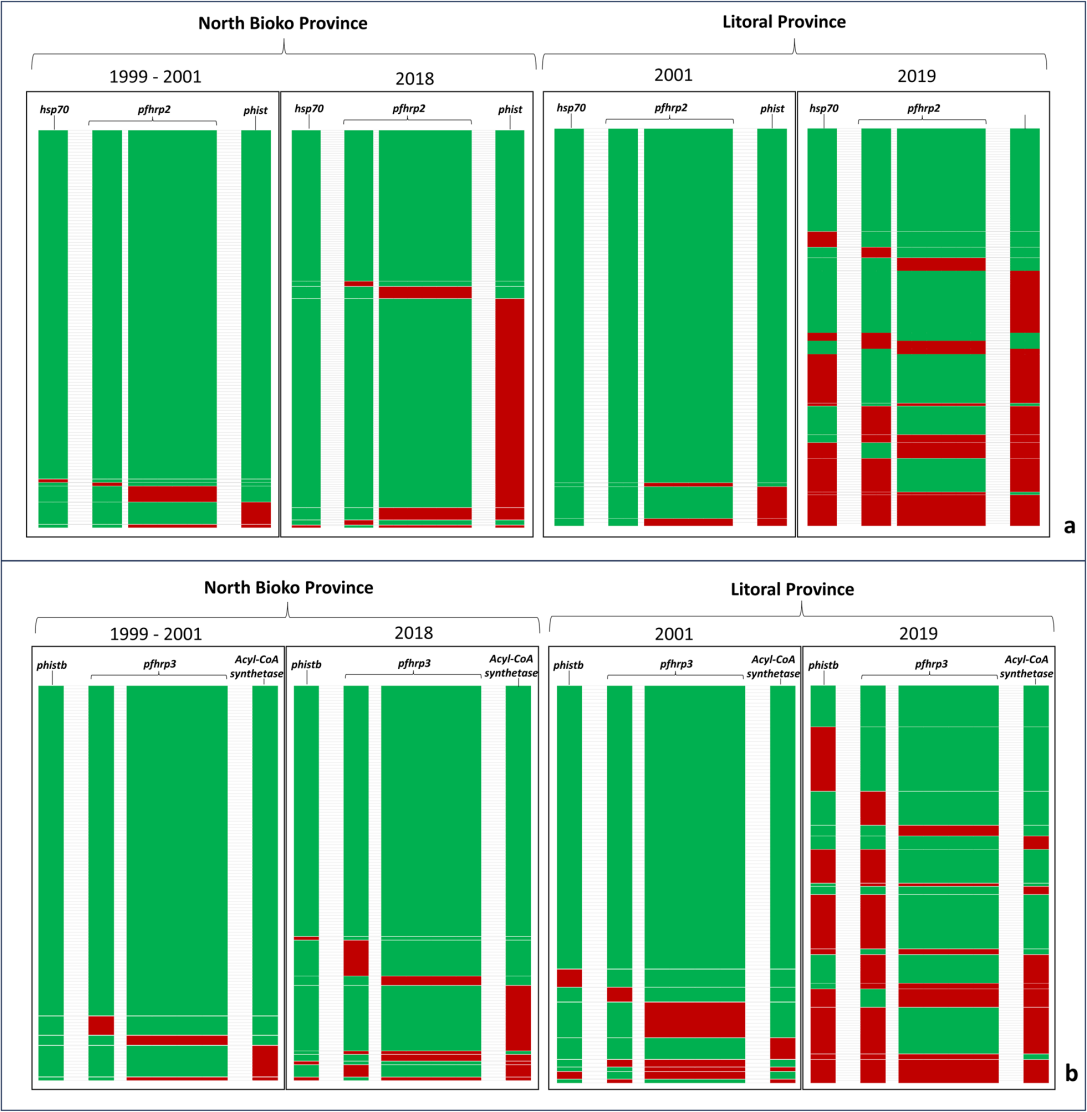
Deletion patterns and combinations in *pfhrp2* and *pfhrp3* genes and their flanking regions per year and locality. Each row represents one sample. Each sample has the information of deletion or presence of an upstream gene, exon 1 and exon 2 of pfhrp2 or pfhrp3, and of the downstream gene. Green means the presence of the genes labelled above, and red means gene deletion. **a** Deletion patterns of *pfhrp2* gene and its flaking genes. **b** Deletion patterns in *pfhrp3* gene and its flanking genes

A higher frequency of deletions was found in downstream flanking *pfhrp2* and *pfhrp3* genes than in upstream. These downstream deletions increased over time reaching frequencies over 15% (Fig. 2; Table S4). Different haplotypes combining *pfhrp2*^*Del*^ and *pfhrp3*^*Del*^ and their flanking genes suggested that the deletions occur independently, being the haplotype with the three genes deleted the least common (Fig. 2).

**Fig. 3:**
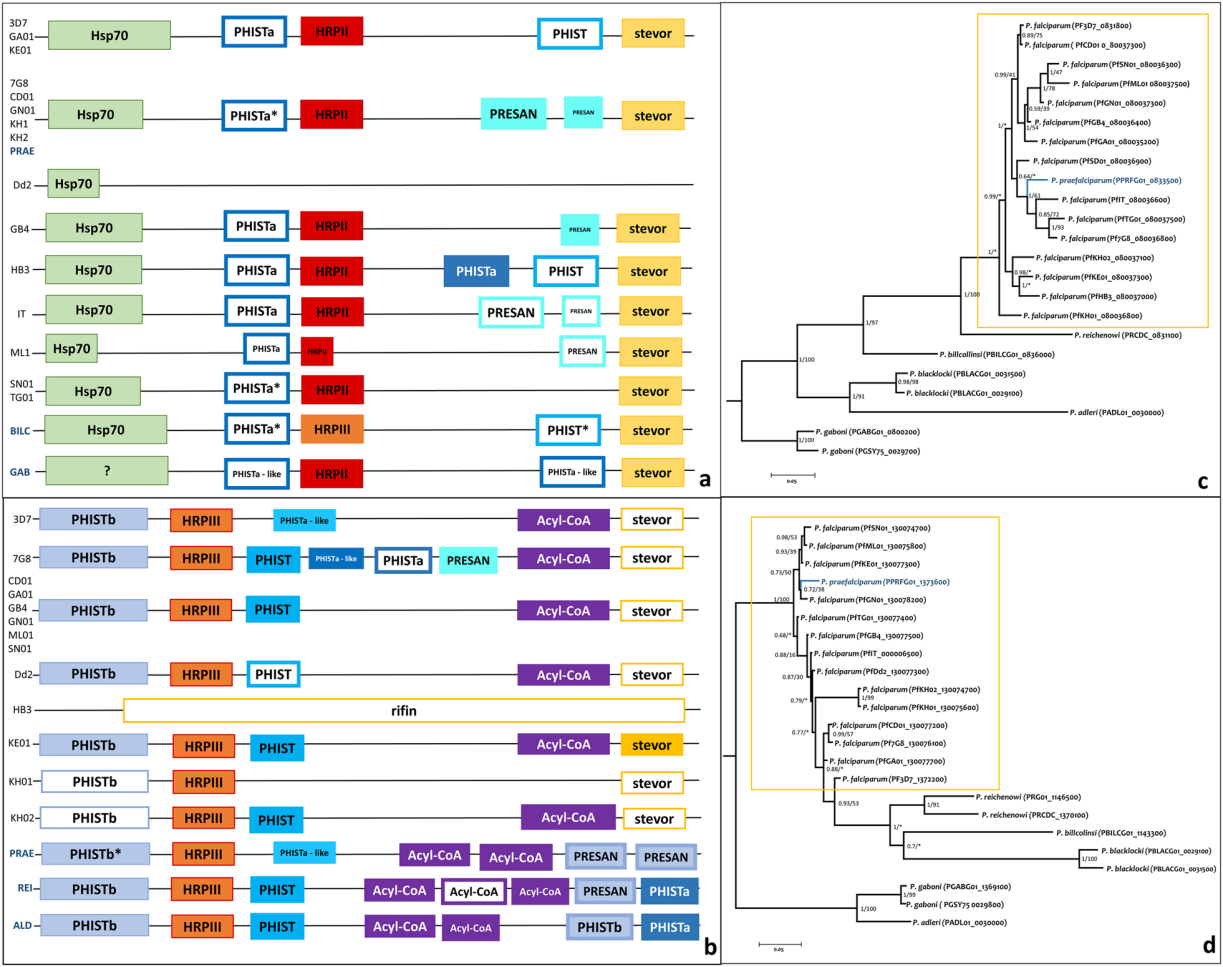
Haplotype diversity and phylogenetic relationships of *hrp*2 and *hrp3* genes of *Plasmodium* species in the subgenus *Laverania.* **a**, **b** Haplotype diversity of *hrp2* and *hrp3* genes respectively, including *P. falciparum* and other *Plasmodium* spp. from subgenus *Laverania* with their region available. Full coloured squares represent genes, and white squares represent pseudogenes. Code before each row is the identification of *P. falciparum* strains and the other *Laverania* species (BILC: *Plasmodium billcollinsi*; GAB: *Plasmodium gaboni*; PRAE: *Plasmodium praefalciparum*; REI: *Plasmodium reichenowi*; ALD: *Plasmodium adleri*). When there is more than one code before the row means that all strains share the same genotype. **c**, **d** Phylogenetic relationship of *hrp2* and *hrp3* genes respectively. Posterior probabilities/bootstrap supports are shown for each node. *Indicate inconsistencies between the Bayesian and Maximum Likelihood methods

High diversity of haplotypes was also found analysing the synteny of these regions among available annotated *P. falciparum* sequences and *Plasmodium* spp*.* from subgenus *Laverania* (Fig. 3a, b). For the*pfhrp2* block, all the species and strains, except Dd2 (with *pfhrp2* deleted), conserved the *hsp70*, *PHISTa* (pseudogene), *hrp2,* and *stevor* in that order, whereas with different gene lengths. However, immediately downstream of *pfhrp2*, they presented different genes from PHIST family in different copy—number (Fig. 3a). Similarly, for *pfhrp3* block, all the *P. falciparum* strains, except HB3 (with *pfhrp3* deleted), conserved *PHISTb*, *hrp3*, *acyl-CoA,* and *stevor* genes (generally as a pseudogene), but between *hrp3* and *Acyl-Coa* there were different number of PHIST family genes or pseudogenes (Fig. 3b). Interestingly, *pfhrp3* block of the other *Laverania* species have more than one *Acyl-CoA* gene copies followed by two PHIST family genes without *stevor* gene.

Figure 3c, d show the phylogenetic relationships among the *Laverania* species for *hrp2* and *hrp3* genes, respectively. It is worth noticing that *Plasmodium praefalciparum* fell within *P. falciparum* clade in both cases, without clear separation from *P. falciparum hrp2* and *hrp3* genes. Additionally, these two phylogenetic trees do not resemble the phylogenetic relationships that have been reported before using other nuclear genes or mtDNA genomes [41].

### Population genetic analyses

More than 50% of samples genotyped had infections with more than one allele in at least one locus (Table S5). Litoral province presented a higher frequency of multiple infections than North Bioko, the difference between provinces was significant for 2001 (p-value = 0.00895; X^2^ = 6.8313), but not for 2018–2019 (p-value = 0.6648; X^2^ = 0.1878). Both regions had opposite temporal trends in the multiplicity of infection, decreasing over time in Litoral province and increasing in North Bioko. However, such temporal differences were not statistically significant (Table S5). All seven neutral microsatellite markers were polymorphic for all samples, ranging in allele number from 1 to 20. Very high heterozygosity was found in all the groups (Table 2).

**Table 2:** Microsatellite fragment size ranges, number of alleles, and heterozygosity (He) per locus, year, and locality

Locus	Allele ranges^a^	No. of alleles^a^	He^a^	Allele ranges^b^	No. of alleles^b^	He^b^
Litoral 2001						
PolyA	130–181	14	0.906	130–181	13	0.898
TAA109	112–208	16	0.845	112–199	13	0.838
TA1	154–187	12	0.899	154–187	12	0.873
PfPK2	157–199	11	0.886	157–187	9	0.852
2490	71–81	7	0.748	77–89	5	0.669
CM313	172–270	27	0.963	172–270	19	0.951
CM383	111–167	16	0.826	123–155	12	0.858
Total (all loci)						0.998
Litoral 2019						
PolyA	120–180	20	0.906	126–180	17	0.891
TAA109	112–196	12	0.809	151–190	9	0.812
TA1	130–187	13	0.877	130–184	12	0.884
PfPK2	145–178	10	0.864	145–178	9	0.869
2490	65–92	8	0.736	65–92	7	0.736
CM313	160–264	30	0.945	160–264	26	0.952
CM383	111–170	22	0.845	119–170	18	0.852
Total (all loci)						0.999
Malabo 2001						
PolyA	108–177	18	0.909	108–177	14	0.918
TAA109	154–199	12	0.823	160–199	8	0.829
TA1	151–193	12	0.865	151–193	11	0.869
PfPK2	160–181	8	0.842	160–181	7	0.835
2490	74–86	4	0.475	74–83	3	0.501
CM313	210–270	26	0.951	210–270	16	0.912
CM383	123–167	21	0.889	125–163	15	0.847
Total (all loci)						0.998
Malabo 2018						
PolyA	120–180	21	0.930	120–180	20	0.922
TAA109	145–208	19	0.888	148–208	15	0.870
TA1	151–193	14	0.884	154–187	11	0.864
PfPK2	151–199	13	0.888	154–199	12	0.867
2490	65–92	9	0.558	65–92	9	0.602
CM313	210–264	26	0.958	212–264	23	0.952
CM383	111–161	19	0.902	119–161	17	0.910
Total (all loci)						0.997

^a^Considering all samples including those where multilocus genotypes could not be phased

^b^Excluding complex infections where multilocus genotypes could not be inferred

### Population structure and origin of deletions

**Fig. 4:**
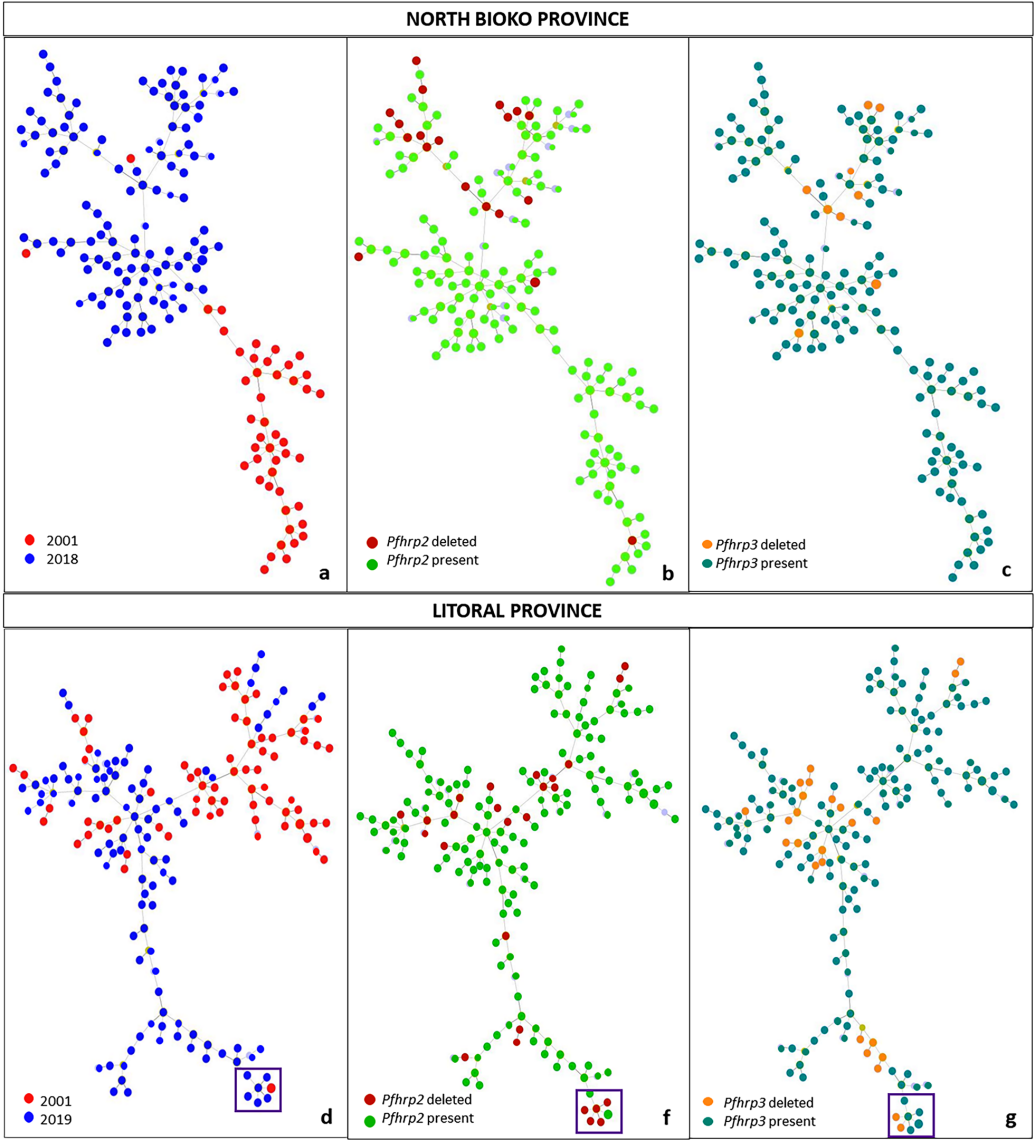
Minimum spanning tree of microsatellite allelic data by goeBURST showing genetic relatedness of *P. falciparum* isolated from Bioko North and Litoral province. Each genotype is represented by a circle, and the colour represents the different characteristic. **a** Tree for all samples from Bioko North province (2001 and 2018). **b** Tree showing the *pfhrp2*^*Del*^ for Bioko North province. **c** Tree showing the *pfhrp3*^*Del*^ for Bioko North province. **d** Tree for all sample from Litoral province (2001 and 2019). **f** Tree showing the *pfhrp2*^*Del*^ for Litoral province. **g** Tree showing the *pfhrp3*^*Del*^ for Litoral province. Purple squares remarks one group of samples that suggest the potential beginning of clonal expansion of parasites with deletions from an ancient sample without deletions

The analysis of genealogical relationships between the samples from North Bioko and Litoral province showed multiple origins of deletions (Fig. 4), for that analysis there were included 430 samples (Table S6). North Bioko province populations from 2001 (in red) and 2018 (in blue) were clearly separated conforming two different clusters (Fig. 4a). Contrastingly, there were different and dispersed origins for *pfhrp2*^*Del*^ and *pfhrp3*^*Del*^, there was not a particular cluster of genotypes with deletions in the minimum spanning tree (Fig. 4b, c). However, some *pfhrp2*^*Del*^ (in red) conformed small groups with more than two isolates. In the case of Litoral province isolates from 2001 and 2019, although some temporal diversification could be observed, the network showed associated isolates from the two populations (Fig. 4d). Also, the network representing gene deletions showed multiple different origins for *pfhrp2*^*Del*^ and *pfhrp3*^*Del*^ (Fig. 4e, f). However, there was a group where 2019—isolates with deletions were grouped with an old non-deleted isolate from 2001 (Fig. 4e, f; purple squares).

### Population structure and genetic relatedness between the two provinces

**Fig. 5:**
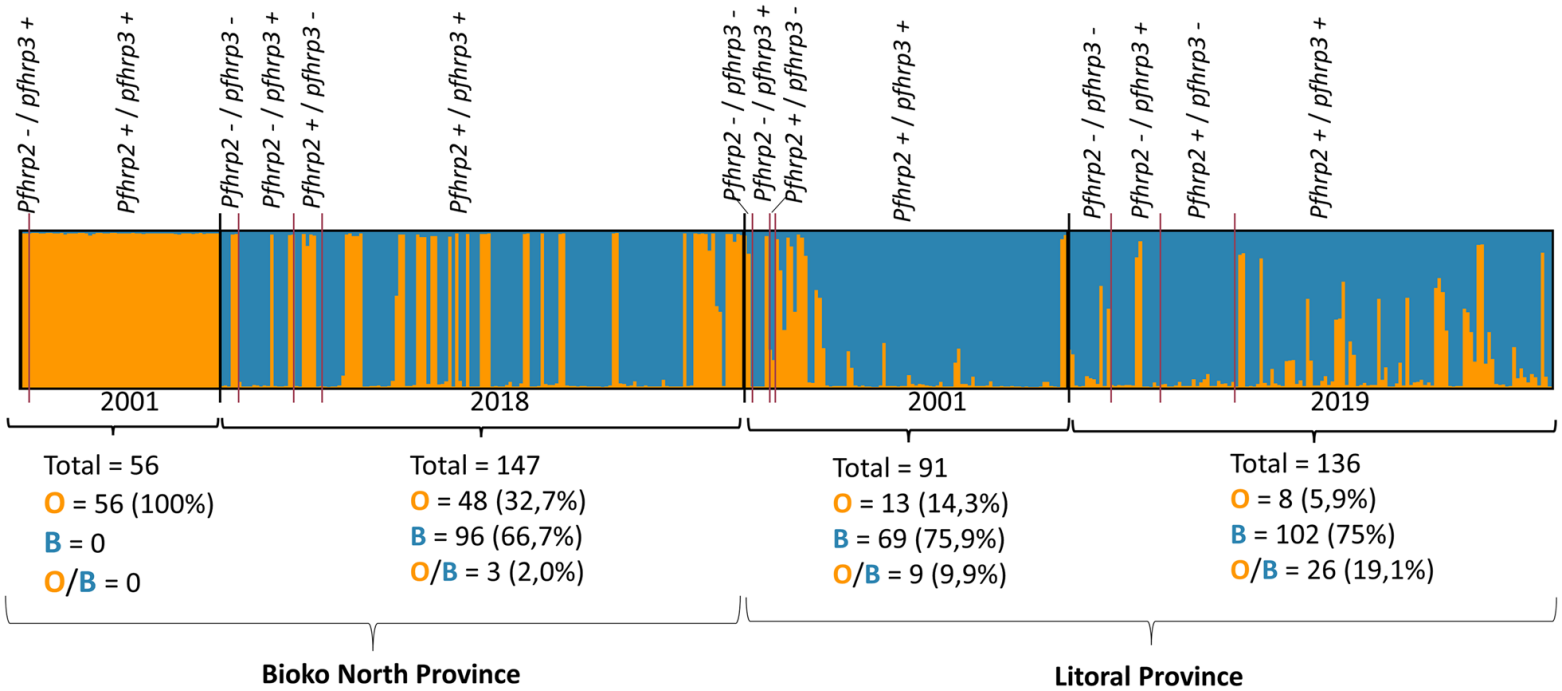
Bayesian cluster analysis of *P. falciparum* samples from Bioko North and Litoral provinces divided per year and *pfhrp2*^*Del*^ and *pfhrp3*^*Del*^ haplotypes (k = 2). Each column represents one sample. Each square below the graph represents number of samples included in each population; O = population represented in orange; B = population represented in blue; O/B = mixed population with percentage of each O and B population between 30 and 70%

Using Structure v.2.3.4, only two clusters were identified for *P. falciparum* samples from Bioko North and Litoral provinces (k = 2, Fig. 5). The only cluster obtained for North Bioko Province samples from 2001 (in orange) was maintained in 2018, however, a second and more predominant cluster (in blue) appeared in 2018. Something to highlight is that the cluster detected in 2018 (in blue) was the main cluster in Litoral province population for both years. Importantly, there were no specific clusters linked to the *pfhrp2*^*Del*^ and *pfhrp3*^*Del*^ gene genotypes. Indeed, different, and independent origins for *pfhrp2*^*Del*^ and *pfhrp3*^*Del*^ deletions were obtained in this study (Fig. S2). However, there are small clusters of 2–5 isolates with single *pfhrp2*^*Del*^/*pfhrp3*^*Del*^ or double deletions.

### Amino acid variation in *pfhrp2* and *pfhrp3* genes

There were successfully analysed 144 sequences of *pfhpr2* and 85 sequences of *pfhrp3* (Table S6). There were no differences in amino acid repeats in the low complexity regions of both *pfhrp2* and *pfhrp3* genes between different years and localities (Table S7; S8). In the case of *pfhrp2*, amino acid repeats 2 (AHHAHHAAD) and 7 (AHHAAD) were the most common, with repeat 2 being the most abundant after RDT introduction, ranging from 1 to 10 or more repetitions per sequence. Although repeats 2 and 7 were present in 100% of samples in most years included in this investigation, North Bioko sequences from 1999 show repeat 19 as the most common (AHHAA, ranging from 0 to 24), whereas 2001 Litoral Province have type 4 (AHH, ranging from 0 to 31) as the most common. *Plasmodium praefalciparum* shows types 2 and 7 as the most common; however, type 4 was the most common in the other species from subgenus *Laverania*, except in *P. adleri* (Table S9). Regarding amino acid repeats found in the *pfhrp3* gene, repeats 7 (AHHAAD) and 16 (AHHAAN) were the most common ones, being in 100% of sequences for all years except 2019. But repeat 7 appeared only one or two times per sequence, while repeat 16 appeared up to 15 times per sequence. In other species, this pattern changes, except *P. praefalciparum*, which also has type 16 as the most common repeat (Table S10).

Similarly, the frequency of epitopes was maintained over time. Specifically, 3A4 and C1-13 appeared in all samples, except in 1999, with a median frequency above 10 times per sequence (Table S9). These two epitopes were also the most common in *P. praefalciparum*, but not in other species (Table S12). In *pfhrp3* gene sequences, only epitopes 3A4 and 1E1-A9 appeared in lower frequency (< 1 per sequence).

## Discussion

This study shows a significant increase of *pfhrp2*^*Del*^ and *pfhrp3*^*Del*^ frequencies in Equatorial Guinea after RDT introduction, being also significant the differences between geographical areas. In addition, multiple origins of deletions were evidenced, probably due to the described instability of the subtelomeric regions [42] as suggested by sequencing and comparative phylogenetic analyses done in this study, without clear clusters of parasites with *pfhrp2*^*Del*^ or *pfhrp3*^*Del*^. Notable, there were no significant differences in amino acid sequences of exon 2 of *pfhrp2* and *pfhrp3* before and after RDT introduction.

The reported high frequency of deletions post-RDT agrees with those from close West and Central African countries [3]. Nevertheless, this contrasts with the lower frequencies reported in bordering countries, such as Cameroon [6, 7] and Gabon [8], whose frequencies are closer to the deletion frequencies found in Equatorial Guinea among samples collected previously to RDT introduction. The high pre–RDT *pfhrp2*^*Del*^ and *pfhrp3*^*Del*^ and the observed temporal increase, considering also the intermediate published *pfhrp2*^*Del*^ prevalence (5.3%) in Litoral province [5], establish Equatorial Guinea as a high-risk country for RDTs losing their efficacy.

One model of *pfhrp2*^*Del*^ selection proposes that in areas with more than 1% starting frequency, the spread of deletions could be significantly faster compared with areas with < 1% starting frequency [43]. Indeed, it is expected a stronger selection pressure driven by an extended RDT use and properly implementation of “test—treat strategies” [44]. However, these results differ from such predictions. North Bioko province showed a lower frequency of deletions, but RDT is more widely used. Given the lower transmission in North Bioko, there could be complex interactions between inbreeding, selection, and the local epidemiology [44–46] affecting the frequencies of deletions. Regarding differences between island and continental regions, human mobility evidenced by parasite migration, even replacement, in population structure analysis, could explain the observed pattern. One hypothesis is that parasites with deletions could be first selected on the Island and then spread in the continental region.

It is worth noting that there could be bias introduced by the patient’s age. Samples from 2019, with significantly higher deletion frequency, were from adults, while the rest were from children. A high percentage of deletions is expected in adults, because they usually have less multiplicity of infection (MOI), which has been associated with the likelihood of detecting deletions [47]. According to methodological limitations, multiclonal infections could underestimate the frequency of deletions because clones with or without deletions in the same infection cannot be separated.

The results provide further that the subtelomeric location of these genes makes it difficult to assess the probability of deletion occurrence and that such events occur multiple times independently of RDT use. Such complexities are evidenced by the variability of these regions, composed by *pfhrp2* and *pfhrp3* and their flanking genes before RDT introduction, and their evolutionary histories when compared with non–human malaria *Plasmodium* spp. that are part of the *P. falciparum* clade. Add to this the possibility that inbreeding during low transmission seasons could facilitate the expansion of deletions even in the absence of selection by the use of RDTs.

The results disagree with previous reports that established that complete deletions of *pfhrp2* and *pfhrp3* genes are more common than partial deletions [30]. However, they agree with the higher frequency of deletions in downstream flanking genes for *pfhrp2* and upstream for *pfhrp3* [14], that have been related to their potential fitness impact [14], but the putative deletions fitness costs remain unsolved. A study suggested fitness cost for parasites with deletion [48] whereas another reported no fitness cost [49]. Perhaps such differences could be explained because one study used HB3 and Dd2 strains [49] with wider deletion blocks, while the other only had *pfhrp2* and *pfhrp3* deleted [49]. Thus, differences could be related to flanking genes [14].

It can be speculated that the rapid evolution of *pfhrp2* and *pfhrp3* genes, also evidenced by the phylogenetic analyses, together with the high reactivity and the lack of strong evidence of fitness cost for the deletions, suggest that HRP2 and HRP3 proteins could elicit a non- protective immune response. However, it is not clear if there is a specific immune response against *pf*HRP2 [50].

Multiple and independent origins of deletions [12] were identified, in agreement with previous reports. Although such lineages could spread as clusters of deletions [16], no evidence was found in this study. Nevertheless, complex dynamics involving multiple origins, the spread of deletions driven by selection, and demography emphasize the need for molecular surveillance to address the impact of deletions in Equatorial Guinea's malaria control efforts [46]. It seems that predictive models that consider such complexities are still needed. Moreover, population structure analysis was performed using only a subset of samples, the ones with simple infections or with multiallelic only for one locus. So, analysis using other techniques that include all samples, especially the multiclonal ones, could give insightful information.

The absence of temporal changes in amino acid diversity supports that this diversity did not have a direct effect on RDT sensitivity [17]. Indeed, the most frequent repeats and epitopes in Equatorial Guinea were in agreement with previous literature [23, 40], so RDTs based on them are recommended.

A problem in *pfhrp2*^*Del*^ and *pfhrp3*^*Del*^ studies is the differences in study designs. In this study, samples from different population groups and without data about RDT results were used, so the impact of these deletions on RDT efficacy was not assessed. However, to our knowledge, this is the first study on *pfhrp2*^*Del*^ and *pfhrp3*^*Del*^ dynamics in Africa that includes pre-RDT samples and comparisons with other *Plasmodium* spp. to understand the evolutionary dynamics of these genes.

## Conclusions

Although there were differences, *pfhrp2*^*Del*^ and *pfhrp3*^*Del*^ were in high frequency in both Equatorial Guinea provinces. Deletion frequencies significantly increased after RDT introduction, however, they were present before RDT use. *pfhrp2* and *pfhrp3* genes and their flanking genes are in a highly unstable genome region where deletions had multiple origins, but clonal expansions of genotypes with deletions were not found in this study. High genetic diversity of *pfhrp2* and *pfhrp3* genes was observed for *P. falciparum* and other *Laverania* species, providing additional evidence of its rapid evolution. The relative frequency of amino acid repeats was maintained over the years and locations in *P. falciparum*. Thus, RDTs are likely to remain effective if the genes encoding the proteins are present. Overall, molecular surveillance of *pfhrp2*^*Del*^ and *pfhrp3*^*Del*^ is necessary to support decisions leading to mitigating the threat that they could pose.

### Supplementary Information

Supplementary Material 1.
