# Supplementary material for: Evolution of pfhrp2 and pfhrp3 deletions in Equatorial Guinea between the pre– and post–RDT introduction
Source: Malar J. 2024 Jul 18;23:215. doi: 10.1186/s12936-024-05036-4 (PMC11264669; doi:10.1186/s12936-024-05036-4)
Supplement: Supplementary file 1 — Supplementary Material 1. [file 12936_2024_5036_MOESM1_ESM.pdf]

Evolution of *pfhrp2* and *pfhrp3* deletions in Equatorial Guinee between the pre – and post – RDT introduction.

Irene Molina - de la Fuente[1,2,3], M. Andreína Pacheco[4], Luz García[2,3], Vicenta González[2,3], Matilde Riloha[5], Consuelo Oki[5], Agustín Benito[2,3], Ananias A. Escalante[4], Pedro Berzosa[2,3]

[1]Biomedicine and biotechnology Department, University of Alcalá, Ctra.Madrid-Barcelona Km.33,600, 28871, Alcalá de Henares, Spain

[2]National Centre of Tropical Medicine, Carlos III Institute of Health, C/ Sinesio Delgado 10, 28029, Madrid, Spain

[3]Consorcio Centro de Investigación Biomédica en Red – CIBERINFEC ISCIII, C/ Sinesio Delgado 10, 28029, Madrid, Spain

[4] Biology Department/Institute of Genomics and Evolutionary Medicine (iGEM), Temple University, (SERC - 645), 1925 N. 12 St, Philadelphia, PA, 19122-1801, USA.

[5]National Programne for Malaria Control, Ministry of Health and Social Welfare (MINSABS), Malabo, Equatorial Guinea

Supplementary material

Figure S1. The Equatorial Guinee map shows the two provinces where samples were collected, Litoral and Bioko Norte.

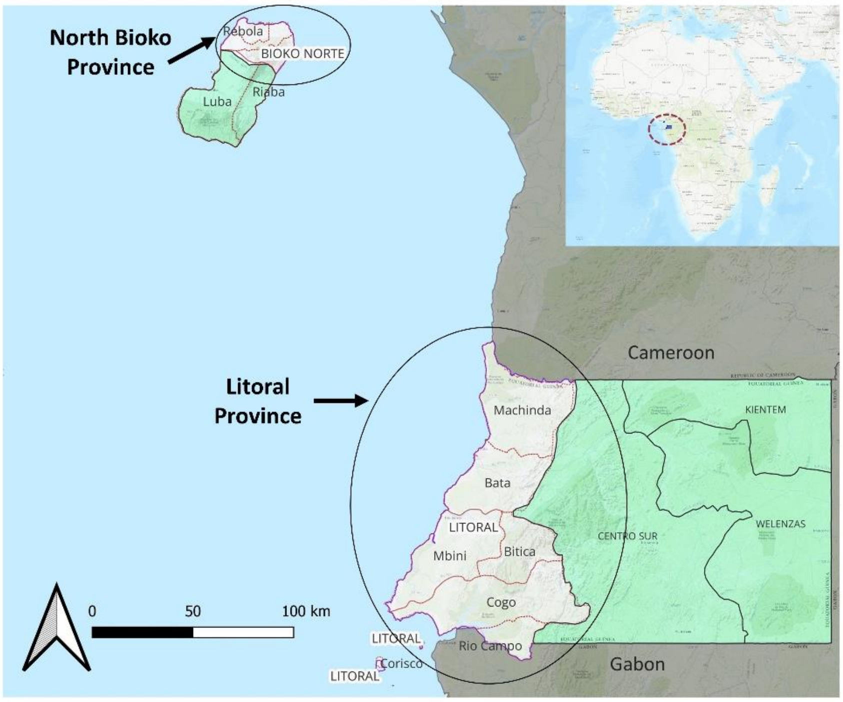

Table S1. Primers for each microsatellite combined with GeneScan350 ROX dye size standard. bp: base pairs of the amplified fragment. Ch: chromosome

Micrt.	Ch	PCR	Primer				bp
			Forward		Reverse		
**Poly A**	6	1º	PolyA-F	AAA ATA TAG ACG AAC AGA	PolyA-R	ATC AGA TAA TTG TTG GTA	159-204
		2º	PolyA-2-F	AAA ATA TAG ACG AAC AGA	PolyA-3IR	***6-FAM-** GAA ATT ATA ACT CTA CCA	
**TA1**	4	1º	TA1-3F	CTA CAT GCC TAA TGA GCA	TA1-R	TTT TAT CTT CAT CCC CAC	114-241
		2º	TA1-2-F	***6-FAM-**CCG TCA TAA GTG CAG AGC	TA1-2-F	TTT TAT CTT CAT CCC CAC	
**PfPK2**	12	1º	PFPK2-F	CTT TCA TCG ATA CTA CGA	PFPK2-R	CCT CAG ACT GAA ATG CAT	
		2º	PK2-2-F	TAG TAA CGA TGT TGA CAA	PK2-2-R	***HEX-**AAA AAG GAG GAT AAA TAC AT	159-192
**TA109**	6	1º	TA109_3-F	TAG GGA ACA TCA TAA GGA T	TA109_3-R	CCT ATA CCA AAC ATG CTA AA	
		2º	TA109-2-F	***HEX -** GGTTAAATCAGGACAACAT	TA109-2-R	CCT ATA CCA AAC ATG CTA AA	154-208
**2490**	10	1º	2490-F	TTC TAA ATA GAT CCA AAG	2490-R	ATG ATG TGC AGA TGA CGA	
		2º	2490-2-F	TTC TAA ATA GAT CCA AAG	2490-2-R	***6-FAM -** AGA ATT ATT GAA TGC AC	75 - 84
**313**	2		313-F :	***6-FAM -** TCCCTTTTAAAATAGAAGAAA	313-R	GATTATATGAAAGGATACATG	225 – 246
**383**	3		383-F	***HEX -** AATAGGAACAAATCATATTG	383-R	AGATATCCAGGTAATAAAAAG	122 - 149

Table S2. Frequency of deletion in exon 1-2 and exon 2 of *pfhrp2* and *pfhrp3* genes by province per year. Significance of differences were assessed using X^2. F= frequency of deletion in percentage.

<table>
<tr><th rowspan="3">Province</th><th rowspan="3">Year</th><th rowspan="3">N</th><th colspan="3">Pfhrp2</th><th colspan="3">Pfhrp3</th></tr>
<tr><th>Exon1-2</th><th>Exon 2</th><th>Any deletion</th><th>Exon 1-2</th><th>Exon 2</th><th>Any deletion</th></tr>
<tr><th>F % (n)
95% CI</th><th>F % (n)
95% CI</th><th>F % (n)
95% CI</th><th>F % (n)
95% CI</th><th>F % (n)
95% CI</th><th>F % (n)
95% CI</th></tr>
<tr><td rowspan="3">North Bioko</td><td>1999 - 2001</td><td>127</td><td>0.79 (1)
0.14 – 4.33</td><td>4.72 (6)
2.18 – 9.92</td><td>5.51 (7)
2.70 – 10.94</td><td>4.72 (6)
2.18 – 9.92</td><td>3.15 (4)
1.21 – 7.82</td><td>7.87 (10)
3.84 – 14.00</td></tr>
<tr><td>2018</td><td>131</td><td>3.88 (4)
1.19 – 7.59</td><td>8.40 (11)
4.75 – 14.41</td><td>11.45 (15)
7.06 – 7.52</td><td>12.21 (16)
7.66 – 18.92</td><td>5.34 (7)
2.61 – 10.62</td><td>16.79 (22)
11.35 – 24.11</td></tr>
<tr><td colspan="2">p – value</td><td>0.385</td><td>0.348</td><td>0.216</td><td>0.054</td><td>0.573</td><td>0.047</td></tr>
<tr><td rowspan="3">Litoral</td><td>2001</td><td>113</td><td>0.0 (0)
0.00 – 3.29</td><td>2.65 (3)
0.91 – 7.52</td><td>2.65 (3)
0.91 – 7.52</td><td>6.19 (7)
3.03 – 12.24</td><td>13.27 (15)
8.21 – 20.75</td><td>17.70 (20)
11.76 – 25.76</td></tr>
<tr><td>2019</td><td>195</td><td>36.41 (71)
29.65 – 43.59</td><td>19.49 (38)
14.17 – 25.75</td><td>43.08 (84)
36.02 – 50.34</td><td>37.95 (74)
31.11 – 45.16</td><td>18.97 (37)
13.73 – 25.19</td><td>50.77 (99)
43.53 – 57.98</td></tr>
<tr><td colspan="2">P - value</td><td>< 0.01</td><td>< 0.01</td><td>< 0.01</td><td>< 0.01</td><td>0.222</td><td>< 0.01</td></tr>
</table>

Table S3. Frequency of each deletion haplotype by province and year. The significance of differences was assessed using an X square. N = number of samples

Province	Year	N	***Frequency of combination in percentage (number of samples)<br>95% Confidence Interval***			
			pfhrp2 - / pfhrp3 -	***pfhrp2 - / pfhrp3 +***	***pfhrp2 + / pfhrp3 -***	***pfhrp2 + / pfhrp3 +***
**North Bioko**	1999 - 2001	127	0.0	4.72 (6)<br>1.75 – 10.0	3.15 (4)<br>0.86 – 7.87	92.13 (117)<br>86.0 – 96.16
	2018	131	1.53 (2)<br>0.16 – 5.41	6.87 (9)<br>3.19 – 12.64	3.82 (5)<br>1.25 – 8.68	87.79 (115)<br>80.92 – 92.85
	p - value					0.4459
**Litoral**	2001	113	0.88 (1)<br>0.02 – 4.83	1.77 (2)<br>0.22 - 6.25	12.39 (14)<br>6.94 – 19.91	84.96 (96)<br>77.01 – 90.99
	2019	195	7.18 (14)<br>3.98 – 11.75	12.31 (24)<br>8.05 – 17.76	11.79 (23)<br>7.63 – 17.17	71.28 (139)<br>64.38 – 77.52
	p - value					< 0.005

Table S4. Frequency of deletions in flanking regions of *pfhrp2* and *pfhrp3* by province and year. The significance of differences was assessed using an X square.

Province	Year	N	Flanking *Pfhrp2*		Flanking *Pfhrp3*	
			heat shock protein 70	*Plasmodium* exported protein (PHIST) - pseudogene	*Plasmodium* exported protein (PHISTb)	acyl-CoA synthetase
			PF3D7_0831700	PF3D7_0831900	PF3D7_1372100	PF3D7_1372400
			F (n)<br>95% CI	F (n)<br>95% CI	F (n)<br>95% CI	F (n)<br>95% CI
North Bioko	1999 - 2001	127	0.79 (1)<br>0.14 – 4.33	6,30 (8)<br>3.23 – 11.94	0,0 (0)<br>0.00 – 2.94	8.66 (11)<br>4.91 – 14.85
	2018	131	0.76 (1)<br>0.02 – 4.18	45.04 (59)<br>36.78 – 53.58	2.29 (3)<br>0.18 – 6.52	22.90 (30)<br>16.54 – 30.81
	p – value		1	< 0.05	0.259	0.003
Litoral	2001	113	0.00 (0)<br>0.00 – 3.29	9.73 (11)<br>5.52 – 16.59	6.19 (7)<br>3.03 – 12.24	7.08 (8)<br>3.63 – 13.35
	2019	195	29.23 (57)<br>22.95 – 36.15	49.23 (96)<br>42.02 – 56.47	50.77 (99)<br>43.53 - 57.98	41.03 (80)<br>34.05 – 48.28
	p – value		< 0.05	< 0.05	< 0.05	< 0.05

Table S5. Genetic polymorphism of infections included. * It refers to monoclonal isolates or isolates with more than one allele only for one locus. That analysis excluded polyclonal infections with more than one allele for more than one locus because using microsatellites it is not possible to know what alleles conform each clone.

Province	Year	Number of isolates	Multiple infection	Single infections	Percentage of multiple infections	95% CI for percentage of multiple infections	Total isolates included for population structure*	Total clones included
**Litoral**	2001	81	62	19	76.5	65.8 – 85.2	44	74
	2019	105	70	35	66.7	56.8 – 75.6	68	105
	p-value (X^2)				0.1907 (1.712)			
**North Bioko**	2001	74	41	33	55.4	43.4 – 67.0	38	51
	2018	105	66	39	62.9	52.9 – 72.1	74	124
	p-value (X^2)				0.3973 (0.7165)			
Overall		365	239	126	65. 5	60.4 – 70.4	150	354

Figure S2. A minimum spanning tree of microsatellite allelic data showing genetic relatedness of *Plasmodium falciparum* populations according to their origin or *pfhrp*2/3 deletion status.

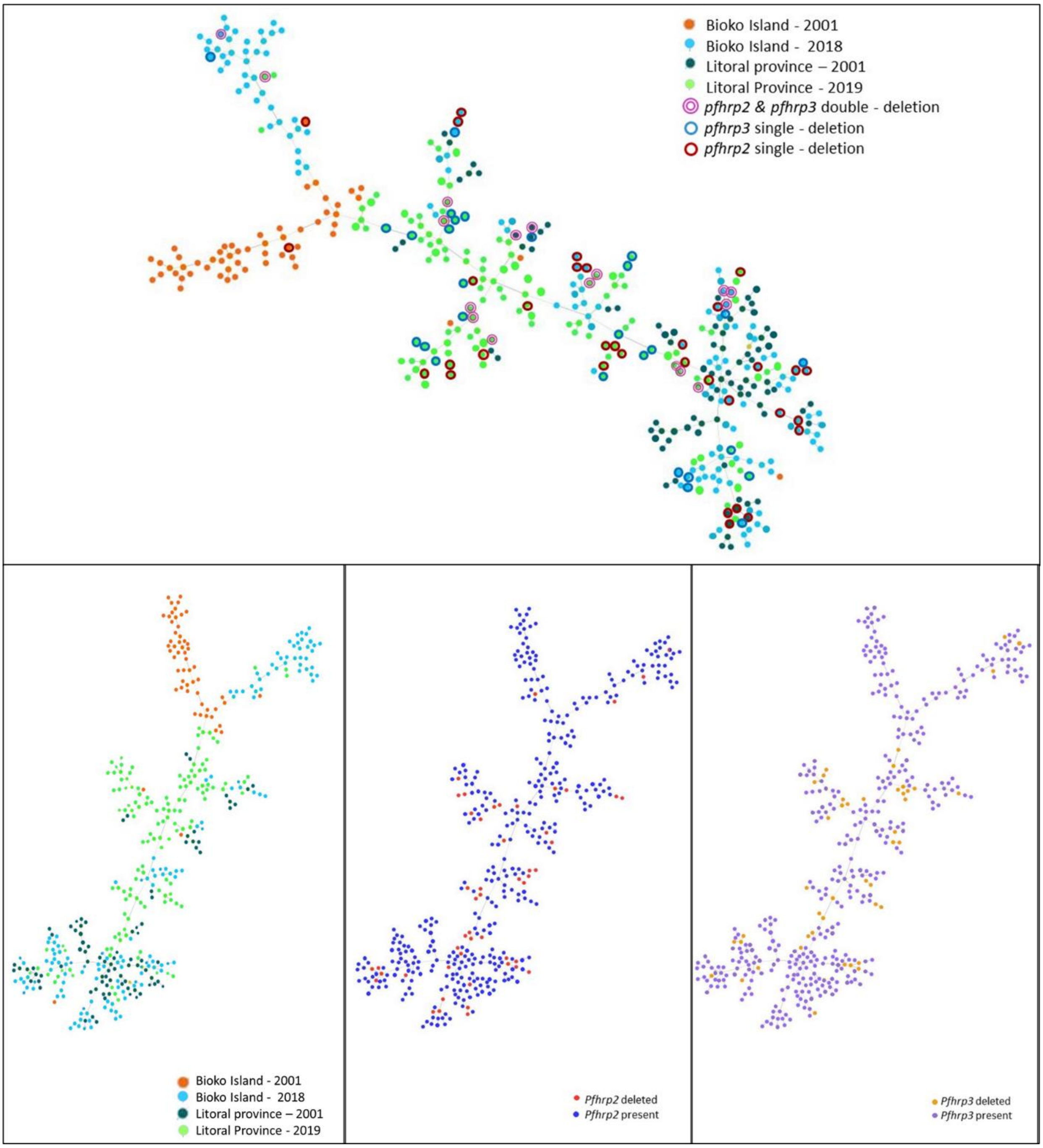

Table S6. Number of samples included for microsatellites analysis and exon 2 genetic analysed by year and province.

**Province**	**Year**	**Aminoacidic analysis**		**Microsatellite analysis**
		Pfhrp2	***Pfhrp3***	
**North Bioko**	1999 – 2001	26	23	56
	2018	26	20	147
**Litoral**	2001	44	19	91
	2019	48	23	136

Table S7. Frequency and occurrence of amino acid repeat types in exon 2 of *pfhrp2* . N: Range of number of repetitions of each amino acid repeat. F = Occurrence of each type of repeat. The repetitions that are not showed were not found in the analysed sequences.

		1999 – 2001		2018		2001		2019	
Types	Amino acid repeat	N	F (%)	N	F (%)	N	F (%)	N	F (%)
Type 1	AHHAHHVAD	0 - 5	50.0	0 - 7	84.6	0 - 5	65.9	0 - 4	52.1
Type 2	AHHAHHAAD	0 - 13	88.5	3 - 18	**100.0**	2 - 15	**100.0**	3 - 15	**100.0**
Type 3	AHHAHHAAY	0 - 2	61.5	0 - 2	80.8	0 - 3	84.1	0 - 3	89.6
Type 4	AHH	0 - 14	76.9	0 - 18	76.9	0 - 31	77.3	0 - 19	85.4
Type 5	AHHAHHASD	0 - 2	61.5	0 - 2	69.2	0 - 2	77.3	0 – 2	62.5
Type 6	AHHATD	0 - 6	73.1	1 - 7	**100.0**	0 - 8	90.9	0 - 6	91.7
Type 7	AHHAAD	0 - 11	88.5	3 - 10	**100.0**	1 - 10	**100.0**	1 - 11	**100.0**
Type 8	AHHAAY	0 - 3	61.5	0 - 2	84.6	0 - 4	93.2	0 - 3	70.8
Type 9	AAY	0 - 1	3.8	0	0.0	0	0.0	0 - 1	2.1
Type 10	AHHAAAHHATD	0 - 2	11.5	0 - 2	53.8	0 - 3	47.7	0 - 2	27.1
Type 13	AHHASD	0 - 2	15.4	0 - 1	77.0	0 - 2	15.9	0 - 3	22.9
Type 14	AHHAHHATD	0 - 2	19.2	0 - 1	3.8	0 - 2	11.4	0 - 1	6.3
Type 19	AHHAA	0 - 24	92.3	0 - 1	42.3	0 - 4	31.8	0 - 3	45.8

Table S8. Frequency and occurrence of amino acid repeat types in exon 2 of *pfhrp3*. N: Range of number of repetitions of each amino acid repeat. F = Occurrence of each type of repeat.

		**1999 – 2001**		**2018**		**2001**		**2019**	
Types	Amino acid repeat	N	F (%)	N	F (%)	N	F (%)	N	F (%)
Type 1	AHHAHHVAD	0 - 5	26.1	0 - 3	75.0	0 - 3	57.9	0 - 3	26.1
Type 4	AHH	0 - 2	78.3	0 - 2	65.0	0 - 2	94.7	0 - 2	78.3
Type 6	AHHATD	0	0	0	0	0 - 1	26.3	0	0
Type 7	AHHAAD	1 - 1	**100.0**	1 - 2	**100.0**	1 - 1	**100.0**	0 - 1	91.3
Type 15	AHHAHHAAN	0 - 1	30.4	0 - 1	80.0	0 - 13	84.2	0 - 1	60.9
Type 16	AHHAAN	9 - 18	**100.0**	8 - 15	**100.0**	0 - 15	**100.0**	0 - 15	95.7
Type 17	AHHDG	0 - 9	95.7	0 - 7	95.0	2 - 10	**100.0**	0 - 7	91.3
Type 18	AHHDD	0 - 3	91.3	0 - 3	95.0	1 - 14	**100.0**	0 - 3	82.6
Type 19	AHHAA	0	0	0	0.0	0 - 1	36.8	0	0
Type 20	SHHDD	1 - 1	**100.0**	0 - 1	95.0	0 - 1	68.4	0 - 1	91.3

Table S9. Occurrence of amino acid repeat types in exon 2 of *pfhrp2*. N: number of repetitions of each amino acid repeat.

		P. praefalciparum	*P. reichnowi*	*P. billcolinsi*	*P. blacklocki*	*P. gaboni*	*P.adleri*
Types	Amino acid repeat	N	N	N	N	N	N
Type 1	AHHAHHVAD	1	0	0	0	0	0
Type 2	AHHAHHAAD	14	**0**	0	**0**	0	**0**
Type 3	AHHAHHAAY	1	0	0	0	0	0
Type 4	AHH	2	12	9	17	26	0
Type 5	AHHAHHASD	0	0	0	0	0	0
Type 6	AHHATD	0	0	0	**0**	0	0
Type 7	AHHAAD	4	**3**	0	**0**	0	**0**
Type 8	AHHAAY	1	0	0	0	0	0
Type 9	AAY	0	0	0	0	0	0
Type 10	AHHAAAHHATD	3	0	0	0	0	0
Type 12	AHHAAAHHEAATH	1	0	0	0	0	0
Type 13	AHHASD	0	0	0	0	0	0
Type 14	AHHAHHATD	1	0	0	0	0	0
Type 16	AHHAAN	0	19	0	0	0	0
Type 17	AHHDG	0	0	0	19	0	0
Type 19	AHHAA	0	5	0	0	0	0

Table S10. Frequency and occurrence of amino acid repeat types in exon 2 of *pfhrp3*. N: Number of repetitions of each amino acid repeat. The repetitions that are not showed were not found in the analysed sequences.

		P. praefalciparum	*P. reichnowi*	*P. blacklocki*	*P. gaboni*	*P. adleri*
Types	Amino acid repeat	N	N	N	N	N
Type 1	AHHAHHVAD	1	1	0	0	0
Type 4	AHH	1	19	17	0	0
Type 5	AHHAHHASD	1	0	0	0	0
Type 7	AHHAAD	1	**7**	**0**	0	0
Type 15	AHHAHHAAN	1	0	0	0	0
Type 16	AHHAAN	10	0	0	0	0
Type 17	AHHDG	6	3	19	0	0
Type 18	AHHDD	2	5	0	0	0
Type 19	AHHAA	0	2	0	0	0
Type 20	SHHDD	1	1	0	0	0

Table S110. Frequency of linear epitopes in exon2 of *pfhrp2* and *pfhrp3.* P = Prevalence Median (n) = Median frequency (n).

MAb	Major epitope	1999		2018		2001		2019	
		P (%)	Median (n)	P (%)	Median (n)	P (%)	Median (n)	P (%)	Median (n)
Pfhrp2									
**3A4**	**AHHAHHA**	92.3	12	**100.0**	14	**100.**0	14	**100.0**	14
**2G12-1C12**	**DAHHAADAHH**	76.9	3	100.0	5	93.2	5	97.9	4
**1E1-A9**	**AHHAHHV**	50.0	0.5	84.6	2	63.6	1	56.3	1
**A6-4**	**HATDAHH**	73.1	1.5	**100.0**	3	88.6	4	95.8	3
**C1-13**	**AHHAADAHH**	92.3	12	**100.0**	16	**100.**0	17	**100.0**	16
**N7**	**DAHHAADAHHA**	73.1	3	**100.0**	5	93.2	5	97.9	4
**PTL-3**	**YAHHAHHA**	76.9	2	96.2	2	95.5	3	95.8	2
**S2-5**	**AHHASDAHHA**	57.7	1	73.1	1	81.8	1	72.9	1
**TC-10**	**TDAHHAADAHHAADA**	30.8	0	80.8	1	61.4	1	64.6	1
**C2-3**	**HAHHAHHAADAHH**	15.4	0	19.2	0	27.3	0	25.0	0
**Genway**	**AYAHHAHHAAY**	0.0	0	0.0	0	2.3	0	2.1	0
Pfhrp3									
**3A4**	**AHHAHHA**	30.5	0	85	1	84.2	1	65.2	1
**1E1-A9**	**AHHAHHV**	26.1	0	80	1	57.9	1	26.1	0

Table S12. Occurrence of linear epitopes in exon2 of *hrp2* and *hrp3* in other species from subgenus *Laverania*. N = number of repetitions of epitopes.

		P. praefalciparum	*P. reichnowi*	*P. billcolinsi*	*P. blacklocki*	*P. gaboni*	*P. adleri*
		N	N	N	N	N	N
hrp2							
**3A4**	AHHAHHA	18	0	NA	7	0	0
**2G12-1C12**	DAHHAADAHH	4	2	NA	0	0	0
**1E1-A9**	AHHAHHV	1	0	NA	1	0	0
**A6-4**	HATDAHH	4	0	NA	0	0	0
**C1-13**	AHHAADAHH	18	3	NA	0	0	0
**N7**	DAHHAADAHHA	4	1	NA	0	0	0
**PTL-3**	YAHHAHHA	2	0	NA	0	0	0
hrp3							
**3A4**	AHHAHHA	2	0	NA	7	0	0
**2G12-1C12**	DAHHAADAHH	0	4	NA	0	0	0
**1E1-A9**	AHHAHHV	1	3	NA	1	0	0
**C1-13**	AHHAADAHH	0	7	NA	0	0	0
**N7**	DAHHAADAHHA	0	3	NA	0	0	0
**S2-5**	AHHASDAHHA	1	0	NA	0	0	0
